# Effect of pH on Zero Valent Iron Performance in Heterogeneous Fenton and Fenton-Like Processes: A Review

**DOI:** 10.3390/molecules23123127

**Published:** 2018-11-29

**Authors:** Fatemeh Rezaei, Davide Vione

**Affiliations:** 1 Department of Environmental Sciences, Faculty of Natural Resources and Marine Sciences, Tarbiat Modares University, Noor 46414356, Iran; fateme.rezaei@modares.ac.ir; 2 Department of Chemistry, University of Turin, Via Pietro Giuria 5, I-10125 Turin, Italy

**Keywords:** heterogeneous Fenton, Fenton-like, Zero valent iron (ZVI), pH, wastewater treatment

## Abstract

Heterogeneous Fenton processes with solid catalysts have gained much attention for water and wastewater treatment in recent years. In the field of solid catalysts, zero valent iron (ZVI) is among the most applicable due to its stability, activity, pollutant degradation properties and environmental friendliness. The main limitation in the use of ZVI in heterogeneous Fenton systems is due to its deactivation in neutral and alkaline conditions, and Fenton-like processes have been developed to overcome this difficulty. In this review, the effect of solution pH on the ZVI-Fenton performance is discussed. In addition, the pH trend of ZVI efficiency towards contaminants removal is also considered in oxic solutions (i.e., in the presence of dissolved O_2_ but without H_2_O_2_), as well as in magnetic-field assisted Fenton, sono-Fenton, photo-Fenton and microwave-Fenton processes at different pH values. The comparison of the effect of pH on ZVI performance, taking into account both heterogeneous Fenton and different Fenton-like processes, can guide future studies for developing ZVI applications in water and wastewater treatment.

## 1. Introduction

The Fenton reaction is widely accepted as a promising method for the degradation of organic and inorganic pollutants in water and wastewater [1,2,3]. This reaction takes place at room temperature and pressure, which helps reduce the treatment costs [2,4]. Moreover, the reaction is fast and requires easy-to-use reagents [5]. The Fenton process can be applied either to decontaminate wastewater so that it meets the standards for safe discharge into natural water bodies, or to minimize the effluent toxicity and enhance its biodegradability, thereby allowing for an efficient biological depuration in municipal wastewater treatment plants [6]. In the classic homogeneous Fenton process, strong oxidant species are primarily produced by reaction of H_2_O_2_ with iron ions (Fe^2+^) in acidic conditions [7]. The homogeneous Fenton reaction has disadvantages because of catalyst consumption and sludge disposal, due to the need to adjust pH after treatment that causes iron precipitation [8,9,10].

To overcome these drawbacks, heterogeneous Fenton and Fenton-like processes have been developed by researchers over the past two decades. In the heterogeneous Fenton reaction, solid iron oxides (Fe_2_O_3_, Fe_3_O_4_, FeO, FeOOH, etc.) are used as Fenton catalysts with H_2_O_2_ [11,12]. Porous materials (e.g., activated carbon, clay, zeolite, multi-walled carbon nanotubes and polymers) can also be used as support for iron oxides [13,14]. In this case, the overall performance of the Fenton reaction can be increased due to the extra cooperation of the catalyst’s support to adsorb pollutant molecules, and to initiate further pathways for radical generation and pollutants decomposition [13,14,15]. In Fenton-like processes, the generation of free radicals and the degradation of contaminants are carried out by using a catalyst together with magnetic field, ultrasound energy, ultraviolet radiation, microwave irradiation, other oxidizing reagents (e.g., persulfate), or a combination of them [16,17].

Among the catalysts used in heterogeneous Fenton and Fenton-like processes, zero-valent iron (ZVI) has attracted wide attention from researchers. ZVI is one of the most promising materials for water and wastewater treatment, due to its cost-effective and environmentally friendly production [18], non-toxicity [19], and ability to efficiently degrade or transform various pollutants such as halogenated compounds [20,21,22], nitrate [23], phosphate [24], polycyclic aromatic hydrocarbons [3,25], heavy metals [23,26,27], arsenic [28], dyes [6,29], and phenol [4,30].

The main factors that affect the performance of ZVI towards contaminants removal in heterogeneous Fenton and Fenton-like processes can be divided into three categories: iron characteristics, operating conditions and solution chemistry [10,11,12]. Understanding the role of these factors is essential to develop the use of ZVI in Fenton and Fenton-like technology, and to adapt the engineering design to site-specific conditions. One of the most important operating parameters in the Fenton reaction is pH [31,32]. The role of pH in controlling Fenton chemistry, free radicals generation, surface charge of catalyst and the amount of dissolved oxygen (DO) in solution has been proven extensively [20,21,33,34,35,36].

Although there are many researches on the preparation of ZVI and its application for the remediation of environmental pollutants, only a few review papers have briefly summarized the effect of pH as an independent parameter on the performance of ZVI in heterogeneous Fenton and Fenton-like processes [9,26,30,37,38,39]. Accordingly, this review comprehensively summarizes the effect of pH on the performance of ZVI for contaminants removal in heterogeneous Fenton and Fenton-like processes. Throughout this work, limitations of ZVI application in heterogeneous Fenton due to pH adjustment needs and the possibility to find alternative solutions are reviewed. In the first section, the general characteristics of ZVI and its use in the removal of contaminants from water and wastewater are discussed. A comprehensive discussion about the effect of pH on the efficiency of ZVI in heterogeneous Fenton processes is provided in the second section. The third section summarizes the effect of pH on different Fenton-like processes based on ZVI. At the end, conclusions and solutions are mentioned to manage the effect of pH on the heterogeneous ZVI-Fenton process aimed at pollutant degradation.

## 2. Zero Valent Iron

Zero valent iron (ZVI) is an effective reagent for the reduction of various environmental contaminants such as metal ions and halogenated organic compounds in water and wastewater, at ambient pressure and temperature [36,40,41]. The removal of contaminants by using ZVI is neither a purely chemical/electrochemical reduction, nor a purely physical adsorption process [10,42]. The process can in fact include complex interfacial pathways such as dissolution, adsorption, redox reaction and precipitation, which can occur simultaneously or sequentially on the iron surface [43,44,45]. ZVI alone is often used as an electron donor (reductant), but in the presence of H_2_O, dissolved O_2_ and, most notably, H_2_O_2_ it becomes an effective source of strongly oxidizing species such as ^●^OH [9,46].

ZVI is not stable in ambient conditions and it converts to Fe^2+^ by reaction with water, dissolved oxygen, or both (Equations (1)–(2)) [10]. Moreover, it can easily get oxidized in the presence of H_2_O_2_ in the framework of the ZVI-Fenton process (Equation (3)) [47]. The generated ferrous ions from ZVI oxidation (Equations (1)–(3)) subsequently participate in the production of free radicals (mainly ^●^OH, Equation (4)). These radicals are responsible for the degradation of organic and inorganic impurities in water and wastewater, causing their transformation and, possibly, organics mineralization (Equation (5)) [20,31,40].

Fe^0^_(s)_ + 2H_2_O_(aq)_ → Fe^2+^_(aq)_ + H_2(g)_ + 2OH^−^_(aq)_ (1)

Fe^0^_(s)_ + O_2(g)_ + 2H^+^ → Fe^2+^_(aq)_ + H_2_O_2(aq)_ (2)

Fe^0^_(s)_ + H_2_O_2(aq)_ → Fe^2+^_(aq)_ + 2OH^−^_(aq)_ (3)

Fe^2+^_(aq)_ + H_2_O_2(aq)_ → Fe^3+^_(aq)_ + OH^−^_(aq)_ + ^●^OH (4)

^●^OH + refractory compounds → H_2_O + CO_2_ + inorganic compounds (5)

Rosales et al. have for instance synthesized ZVI from iron salts by using herbal extracts as reducing agents (rooibos, lemon verbena and camphor), to achieve Fenton degradation of dyes in wastewater [48]. From their results, synthesized rooibos-ZVI removed 90% of dyes in the Fenton reaction within 60 min [48]. Wu et al. have explored the activity of ZVI supported on kaolinite (ZVI-K) for the Fenton degradation of nitrobenzene [13]. The results indicated that ZVI-K could effectively remove nitrobenzene from water (92% degradation under optimal experimental conditions) at pH values of up to 7.2. They proposed that in the first stage of the treatment, degradation was caused by nitrobenzene reduction by ZVI-K followed by heterogeneous Fenton. In the second stage the homogeneous Fenton reaction occurred in the presence of leached Fe, with enhancement of degradation because of the synergistic effect between Fenton reaction and reduction by ZVI-K [13]. A study on cosmetic wastewater treatment with ZVI showed that, while COD removal <15% was obtained by using ZVI or H_2_O_2_ alone, the ZVI-Fenton process (ZVI + H_2_O_2_) removed 84% of COD after 120 min [6]. Likewise, Segura et al. have applied the ZVI-Fenton process for the treatment of drug manufacturing plant wastewater [3], achieving 60% of TOC mineralization. Moreover, biodegradability of wastewater (in terms of the BOD_5_/COD ratio) increased to up to 0.35 from an initial value of 0.18 [3]. Naldoni et al. have studied the degradation of sodium dodecyl benzene sulfonate (DBS) and dodecylpyridinium chloride (DPC) using ZVI as a catalyst in the heterogeneous Fenton reaction [22]. The results showed 51% and 87% mineralization of, respectively, DBS and DPC by using ZVI [22].

In the absence of H_2_O_2_ the removal of water and wastewater contaminants by ZVI can occur through adsorption, reduction and co-precipitation, which is important especially in the case of heavy metals [42,43,44,49]. Moreover, ZVI can also produce H_2_O_2_ and trigger the Fenton reaction starting from dissolved O_2_ (reactions (1-4)), even in the absence of H_2_O_2_ addition. The use of ZVI in the sequestration process for contaminants removal from water and wastewater has been studied widely, exploiting adsorption, reduction and co-precipitation phenomena (e.g., As(V) [42], Cr(VI) and Sb(III) [43], Cd(II) [49], Cu^2+^, CrO_4_^2^^−^, 2-chloroacetophenone, 2,4,6-trinitrotoluene, carbon tetrachloride, trichloroethene [50], NO_3_^−^ and NO_2_^−^ [23,50]). An electron energy loss spectroscopy (EELS) analysis of ZVI has shown that surface-bonded OH groups (Fe−OH) are generated on the ZVI surface in the presence of water, and that they provide effective exchange sites for pollutants in the sequestration process [42]. The unique core-shell structure of ZVI includes a core of metallic iron, a shell of iron (II, III) oxides mixtures near the core, and an iron oxide layer (mostly Fe(III)) near the interface between the particles and the aqueous medium. The exact composition and characteristics of the iron oxide shell depends on the synthesis method and the environmental conditions [19]. The behavior of the ZVI shell is strongly related with the pH of the point of zero charge (pH_PZC_), which corresponds to the pH value at which the oxidized surface is uncharged [51]. At pH values lower than the pH_PZC_, the ZVI surface is positively charged and it can adsorb anions simply via electrostatic attraction. In contrast, at pH > pH_PZC_ the negatively charged surface of ZVI attracts and adsorbs positively charged species, while at the same time one has electrostatic repulsion between anions and the ZVI surface [42,50]. It has been reported that the pH_PZC_ of different iron oxides is typically in the range of 6–9.2 (pH_PZC_ of α-Fe_2_O_3_ = 6.8–9.2, Fe_3_O_4_ ≈ 7.8, α-FeO(OH) = 9, γ-Fe_2_O_3_ = 6.3) [43]. For instance, Cao and colleagues have reported that ZVI could adsorb more tetracycline at pH < 5 than at pH 10 [52]. The authors attributed this result to the occurrence of high amounts of Fe_2_O_3_, Fe_3_O_4_ and FeO(OH) species on the ZVI surface at pH < 5, together with a strong electrostatic attraction between positively charged iron oxides and negatively charged tetracycline molecules [52].

**Figure 1:**
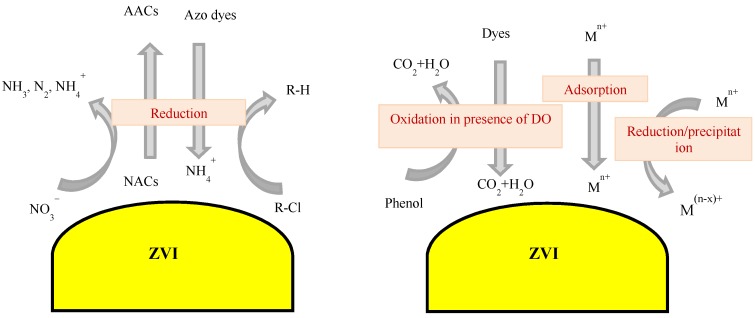
Schematic of pollutants removal by ZVI in the absence of H_2_O_2_. NACs = nitroaromatic compounds; AACs = aminoaromatic compounds.

The standard reduction potential of ZVI (Fe^2+^/Fe^0^) is −0.44 V, which is lower than that of many pollutants (e.g., Cu, Hg, Pb, Ni) [7,18]. Therefore, several metals occurring as water-soluble ions can react with ZVI as an electron donor and precipitate, following reduction into insoluble metal forms. Ling and Zhang [42] and Miehr et al. [49] have also proposed that the removal of inorganic anions (e.g., CrO_4_^2^^−^ and NO_3_^−^) from water by using ZVI, in the absence of hydrogen peroxide, involved anion adsorption to the oxide groups occurring on Fe^0^. In contrast, cations (e.g., Cu^2+^) were removed through reduction on comparatively oxide-free metal. A schematic of the reactions of ZVI with different types of contaminants is presented in Figure 1.

Yoshino et al. have studied the removal of Cu from acidic aqueous solutions by using ZVI [53], and batch experiments were carried out to investigate the effect of pH (2–5) on the Cu removal performance. The pH range was chosen to avoid formation and precipitation of Cu(OH)_2_ at pH > 6. Complete elimination of 1.57 mM Cu was obtained in 35, 30, 20 and 40 min at an initial pH value of 2, 3, 4 and 5, respectively. The authors reported that the pH of the studied systems quickly increased to around 6, except for the case of initial pH 2. At initial pH 3–5, the pH increase could be accounted for by fast iron dissolution accompanied by H^+^ consumption. Due to higher initial [H^+^], the same phenomenon at pH 2 only caused a slight pH increase from 2 to 2.3 after 80 min of reaction. The authors suggested that the most important mechanism in Cu removal was the reduction of Cu^2+^ by ZVI in acidic conditions [53]. Qin et al. have suggested that the Cu^2+^ removal with ZVI/O_2_ involved Cu^2+^ reduction by Fe^0^ at pH 4 (Equation (6)), while the removal mechanism partially changed from reduction to adsorption under adequate dissolved oxygen values at pH 6 [54].

Fe^0^ + Cu^2+^ → Fe^2+^ + Cu^0^ (6)

At pH 6, the concentration of Cu^2+^ was also found to decrease because of deposition processes on the ZVI surface [54]. This hypothesis was confirmed by the XRD patterns of ZVI after reaction at pH 7 that showed copper oxide peaks, whereas only peaks of Cu^0^ could be found in the corresponding XRD pattern of ZVI at pH 4 [55]. According to Jiang et al., about 40% Cu removal was obtained at pH 4 after 120 min, while negligible Cu removal was observed at pH 6 at the same time scale [56]. The partial disagreement between different studies may suggest that the reductive pathway is more robust than that based on adsorption.

Bae and Hanna have reported that high pH values (8–9) could improve the reduction rate of 4-nitrophenol when using low amounts of nano-ZVI (0.010–0.075 g/L), in both un-buffered and buffered suspensions [57]. Their results also suggest that the contaminant reduction rate decreased at pH 6–7 in an un-buffered system, while buffered pH systems could preserve the ZVI particles against dissolution even at high pH values (8–9). These findings highlight the potential impacts of buffering agents on the performance of ZVI [57]. Alowitz and Scherer have shown that Cr(VI) could be removed from water in three steps: (1) adsorption of Cr(VI) to the iron oxides associated with Fe^0^; (2) reduction of Cr(VI) to Cr(III), and (3) co-precipitation of Fe(II/III)/Cr(III) oxy-hydroxides [23]. In this case, alkaline pH can decrease the ZVI performance because it reduces iron solubility and generates a passive oxide layer [23]. On the other hand, different studies have shown that the decrease of pH has contrasting effects on the ZVI performance in the absence of H_2_O_2_. On the one side the acidic pH enhances iron corrosion, but on the other side the acceleration of ZVI corrosion decreases the sites available for contaminant adsorption [53,54,56]. Therefore, the optimal pH conditions for contaminants removal by using ZVI in the absence of H_2_O_2_ depend on the intrinsic characteristics of the contaminant and on the mechanism of its removal [23]. Comprehensive details of mechanism, kinetic parameters, benefits and application of ZVI in the presence and absence of H_2_O_2_ for water and wastewater treatment are already available (see [10,18,20,45,58,59,60,61,62,63,64,65,66], and references therein).

## 3. Effect of pH on ZVI Performance

### 3.1. Heterogeneous Fenton Process. (ZVI-Fenton)

In addition to the iron intrinsic properties, some operational parameters including pH [25,29], dissolved oxygen [67,68], iron dosage [24,69], iron pretreatment [70], ratio of H_2_O_2_/Fe^0^ [2,71] and temperature [41,72,73] can also affect the performance of ZVI in the Fenton reaction, as well as the fate and transport of contaminants. Among the above-mentioned parameters, pH is one of the most important because it directly affects ZVI corrosion and the amount of dissolved oxygen in natural waters and industrial wastewaters [25,29,39,74]. Moreover, pH controls the generation of ions and free radicals in the Fenton reaction [62,74]. Donadelli et al. have studied the efficiency of the ZVI-Fenton removal of the azo dye acid black 1 at pH 3–5, and the dye oxidation was significantly pH-dependent [62,75]. The generation of highly reactive ^●^OH radicals was enhanced in acidic media, and it consequently enhanced the oxidation of azo bonds and aromatic structures [29]. At pH 3 both dissolved Fe(II) and Fe(III) levels initially increased, although Fe(III) started decreasing after 10 min reaction time. The rapid consumption of H_2_O_2_ was probably responsible for the inhibition of the Fe(II) oxidation to Fe(III) (Equation (4)). At pH 5, the main iron species in solution was Fe(III) and the level of Fe(II) was at least two orders of magnitude below that observed at pH 3. As a result, both ZVI corrosion and the ^●^OH radicals production increased with decreasing pH [29].

Ling et al. have studied the degradation of acesulfame (a typical compound that is recalcitrant to biological water treatment) using ZVI-Fenton at pH 6.5 [76]. To examine the mechanism of acesulfame decomposition, two suspensions at 500 and 5000 mg/L ZVI were prepared, and the degradation kinetics of acesulfame and H_2_O_2_ were compared with and without the presence of 2,2’-bipyridine (BPY) 5 mM. BPY can form a complex with Fe^2+^_(aq)_ and block the Fenton reaction, without interfering with the ZVI corrosion chemistry. The H_2_O_2_ degradation rates were not significantly affected by BPY in solution, while 5 mM BPY almost completely inhibited the degradation of acesulfame. The suggested explanation was that H_2_O_2_ decomposition occurred primarily via reduction by ZVI, but acesulfame degradation was due to Fenton-generated ^●^OH radicals [76]. The cited authors also compared the efficiency of pre-oxidized ZVI, prepared by treating ZVI in 10 mM H_2_O_2_ and 1 mM Cl^−^ for 90 min, with untreated ZVI in the same conditions. The pre-oxidized ZVI decomposed 19% of the initial H_2_O_2_, compared to nearly 0% by untreated ZVI. The degradation of H_2_O_2_ by pre-oxidized ZVI was attributed to the heterogeneous Fenton reaction at the iron oxyhydroxide surface, thereby suggesting that pre-oxidized ZVI could be more active than untreated ZVI at neutral pH [76].

Zhang et al. have studied the ZVI-Fenton removal of norfloxacin [77], which is strongly pH-dependent. The best degradation efficiency was observed at pH 3–4, where almost 90% of norfloxacin was removed within 10 min. By increasing the solution pH to around 6, the removal efficiency was reduced to 15% in 40 min. Khan et al. have studied the effect of the initial pH (4–10) on azo-dye removal by ZVI-Fenton [78]. They have found that over 75% of the dye was eliminated in 250 min at pH 4, while removal dropped down to 54% at pH 10. It was concluded that degradation was enhanced in acidic and circumneutral conditions compared to alkaline ones, presumably because of higher ZVI corrosion and lower surface passivation at the lower pH values [78]. These results are in agreement with those of Harada et al. [47], who studied the effect of the solution pH (3–7) on the performance of micro- and nano-sized ZVI in wastewater treatment. The cited authors found a 56% decrease in ^●^OH production by increasing pH from 3 to 4, and a further decrease when raising the pH to 5 [47]. Yuan et al. have shown that the COD removal efficiency of ZVI-Fenton increased rapidly (from 1.3% to 62.4%) by decreasing the initial pH from 9 to 2.5, while a further pH decrease from 2.5 to 2 had little effect on COD removal [79]. The lower pH values may favor the dissolution of passivating species (e.g., ferrous hydroxide) and other protective layers depositing on the surface of Fe^0^, thereby maintaining the number of active sites. The Fenton-like reactions in the presence of dissolved oxygen and without H_2_O_2_ could be enhanced as well at low pH [79]. A similar conclusion has been reached by Cheng et al., who also suggested that weaker and more selective oxidants like FeO^2+^ can be produced at higher pH (Equation (7)). The observed increase in pH as the reaction progressed might explain the fact that ZVI underwent gradual deactivation [80].

Fe^2+^ + H_2_O_2_ → FeO^2+^ + H_2_O (7)

Martins et al. have studied the ZVI-Fenton performance in the treatment of simulated and actual olive mill wastewater [4], analyzing the amount of Fe leached in solution at different pH values. In the pH interval 2–7 the maximum concentration of leached Fe^2+^ was observed at pH 3, and a sharp [Fe^2+^] drop was reported between pH 3 and 4 [4]. Shen and coworkers have reported a decrease in the ZVI-Fenton performance at pH < 3, presumably due to a combination of factors (including the accumulation of H_2_ bubbles at the ZVI-solution interface) [81]. Katsoyiannis et al. have studied the effect of pH (3–11) on the ZVI-Fenton oxidation of As(III) in aerated water [82]. Their results showed that highly bioavailable As(III) (1 mg/L) could be completely converted into less bioavailable As(V) within 1 h by 0.1 g/L ZVI at pH 3. In contrast, by increasing the initial pH to 5 and above, little dissolved As(V) could be detected even after 3 h. These results may be attributed to the fact that, when increasing the pH values, Fe^0^ becomes unstable and reacts with water to form Fe^2+^. The Fe^2+^ ions may hydrolyze to Fe(OH)_2_ or be oxidized to Fe(III) by oxygen, without producing reactive species. [82]. Anyway, it is quite clear that the ZVI-Fenton performance for As(III) removal is pH-dependent [82]. Likewise, Song and Carraway have reported that the removal of 1,1,2,2-tetrachloroethane by nanosized ZVI decreased when increasing the pH from 6.5 to 9.0 [83].

Overall, it can be concluded that the maximum efficiency of ZVI-Fenton toward pollutant degradation can be achieved at pH 3–4, regardless of the target substrate [8]. The advanced explanation(s) may vary in different studies, but enhanced ZVI dissolution at acidic pH and the fact that maximum ^●^OH generation in the Fenton reaction takes place at pH 3 can be regarded as reasonable hypotheses to account for these results [9,46]. Table 1 summarizes some illustrative works carried out on heterogeneous Fenton by using ZVI, together with their main results.

### 3.2. Fenton-Like Processes

As suggested above, the removal of contaminants by the ZVI-Fenton reaction may be limited to acidic conditions [22,82,92]. To overcome this problem, Fenton-like processes have been developed [93]. Depending on the site of the catalytic reactions, these can be classified into heterogeneous Fenton-like and homogeneous Fenton-like processes [46]. In a heterogeneous Fenton-like reaction, Fe^2+^ is replaced by a solid iron catalyst. In contrast, homogeneous Fenton-like processes include a combination of other metal ion(s)/metal ion-organic ligand complexes and H_2_O_2_ [31,94]. However, a more common classification of Fenton-like processes is based on the generation method of ^●^OH radicals. On this basis, Fenton-like techniques include magnetic field assisted-Fenton, sono-Fenton, photo-Fenton, microwave-Fenton, and the use of oxic solutions or other oxidizing reagents (e.g., persulfate) in addition to the catalyst [16,17,67,75]. This latter definition of a Fenton-like process is used in the present review paper. The present section discusses the effect of pH on the performance of different Fenton-like processes that use ZVI as catalyst.

#### 3.2.1. ZVI in Oxic Solutions

These processes make use of ZVI + O_2_ to generate the Fenton reactants (see Equations (1), (2) and (4), avoiding the addition of H_2_O_2_. Yamaguchi et al. have studied the effect of pH on the efficiency of a ZVI/Cu bimetallic catalyst for the Fenton-like treatment of oxic wastewater [25]. The cited authors reported that both dissolution of deposited Cu and ZVI corrosion actively occurred at pH 3, which subsequently led to the in-situ production of Fenton reagents (i.e., Fe^2+^ and H_2_O_2_) and to ^●^OH radical generation [25]. Guan et al. have suggested that the ZVI/O_2_ system is not a useful remediation method, due to the low yields of oxidizing species that can react with organic compounds [93]. In contrast, Joo et al. have studied the degradation of the carbothiolate herbicide molinate in oxic solutions using nano-ZVI [95], and reported over 60% molinate degradation at pH 8.1 (150 min reaction time, 21.4 mM ZVI). The degradation percentage reached 65% at pH 4, under otherwise identical conditions as those used at pH 8.1 [95]. At pH 8.1, accumulation of Fe^2+^ in solution was insignificant due to its rapid oxidation by H_2_O_2_. By decreasing the initial pH value from 8.1 to 4 the concentration of ferrous iron decreased more slowly over time, as a consequence of the balance between released Fe^2+^ from the Fe^0^ surface, and the subsequent Fe^2+^ oxidation to Fe(III) species by H_2_O_2_. Interestingly, the time trend of molinate degradation was relatively similar at both pH 4 and 8.1. The cited authors proposed that the continuous generation of fresh reactants (Fe^2+^, oxygen species, ferryl species and H_2_O_2_) accounted for the process effectiveness at high pH, which was limited by Fe^0^ availability [95]. It could be concluded that the slow release/formation of key reactants and the continuing effectiveness in contaminant degradation could extend the pH range of the possible applications of ZVI-mediated oxidative processes [73,95].

#### 3.2.2. Magnetic-Field Assisted Fenton Process

Zhou et al. have applied magnetic field (MF) as well as ZVI/EDTA for the removal of diclofenac from wastewater [96]. They reported that acidic conditions were more favorable to contaminant removal, but the MF/ZVI/EDTA system could effectively decompose diclofenac in the pH range of 4 to 7 [96]. Different studies have shown that application of magnetic field together with EDTA could accelerate pitting corrosion on the disclosed fresh Fe^0^ sites, in neutral or even alkaline systems [96,97]. The mechanism of contaminant removal by ZVI/EDTA at acidic pH involves the formation of complexes between EDTA and Fe ions (Equation (8)), together with the reaction of the Fe^II^EDTA complex with soluble oxygen (Equation (9)) to yield O_2_^●−^. The latter produces H_2_O_2_ by disproportionation, which finally gives ^●^OH that induces pollutant degradation.

Fe^III^EDTA + e^−^ → Fe^II^EDTA (8)

Fe^II^EDTA + O_2_ → Fe^III^EDTA + O_2_^●^^−^ (9)

Zhou and colleagues have proposed that the magnetic field does not change the mechanism of diclofenac removal by ZVI/EDTA, but that it enhances iron corrosion in a broad pH range (4 to 7) [96]. Pan et al. have studied the degradation of orange G by using ZVI/H_2_O_2_ at different pH values (3–9) in a salty wastewater [98]. They have found that the contaminant removal rate decreased by 50% when the initial pH was increased from 3 to 9. To enhance degradation, the cited authors examined the efficiency of MF/ZVI/H_2_O_2_ in the same conditions. Results showed that the degradation percentage of orange G at pH 9 after 60 min reaction time was increased from 16.4% with ZVI/H_2_O_2_, to 32.7% with MF/ZVI/H_2_O_2_ [99]. Liang et al. [100] and Feng et al. [101] have reported similar results for the removal of chromate and selenite using ZVI and magnetic field. In the presence of a magnetic field, free electrons and Fe^2+^ ions are continually released from reactive anode Fe^0^ sites, and then they are transferred to the surface of Fe_x_O_y_ cathode sites to yield •OH for the oxidation of contaminant molecules. Additionally, reductive adsorption of contaminants could directly occur on the Fe^0^ surface. Some studies have reported that in MF/ZVI systems, ^●^OH radicals could be generated at anodic sites upon oxidation of water even at neutral and alkaline pH values [97,99,100].

**Figure 2:**
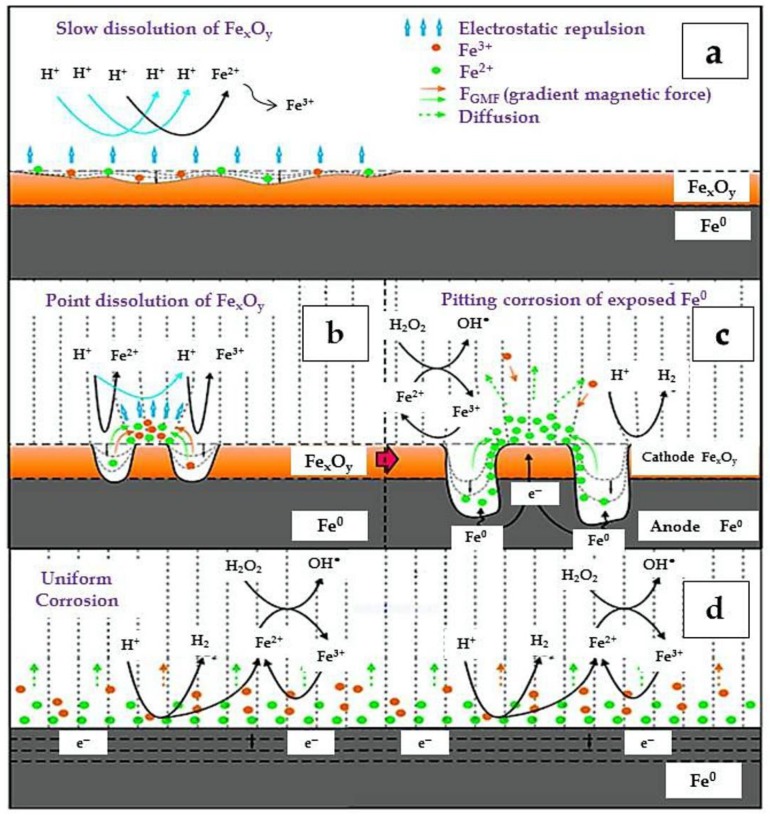
Schematic of a typical MF-ZVI-Fenton system. (**a**): Slow Fe_x_O_y_ dissolution in the ZVI-Fenton system; (**b**): rapid point dissolution of Fe_x_O_y_ in the presence of magnetic field followed by (**c**): pitting corrosion of the exposed Fe^0^ sites and (**d**): uniform corrosion of the ZVI surface. Reproduced with permission from Reference [97].

Xiong et al. have studied the effect of pH (3–10) in a persulfate/ZVI system coupled with magnetic field for the removal of orange G [100]. The application of the magnetic field enhanced orange G degradation by 5–28 folds in the pH range 3–9. However, at pH 10 the removal of the contaminant was significantly inhibited. In this case, the formation of a passive iron oxide layer on the ZVI surface would slow down Fe^0^ corrosion [100]. Still, in the other conditions the solution pH was not a limiting factor for the ZVI performance in the presence of a magnetic field. Moreover, the magnetic field could increase the generation of sulfate radicals and of Fe^3+^ by accelerating the release of Fe^2+^ from the ZVI surface, thereby increasing the rate of the reaction between Fe^2+^ and persulfate. The generated sulfate radicals could degrade orange G, while the Fe^3+^ hydrolysis would yield H^+^ and cause a sharp pH drop. The pH decrease could further increase the ZVI corrosion, thereby amplifying the influence of the magnetic field [100]. Several studies have in fact demonstrated that the main synergistic role of the magnetic field in the system operates through increased surface dissolution and corrosion of ZVI, which occurs together with a decrease in solution pH [97,99,101]. Due to enhanced dissolution, the MF-ZVI-Fenton system can still be operational at neutral and alkaline pH values. A schematic of the MF-ZVI-Fenton process is shown in Figure 2.

#### 3.2.3. Sono-Fenton Process

Man and coworkers have used ultrasound and a ZVI/EDTA/air system (no H_2_O_2_ added) to remove polycyclic aromatic hydrocarbons (PAHs) from textile dyeing sludge, at different initial pH values (3–9) [99]. The reported PAH removal efficiencies showed a slight difference by changing pH from 3 to 6, but they declined significantly by further increasing pH to 9 because of limited formation of Fe^2+^ and Fe^3+^. The reported results suggest that the ultrasound/ZVI/EDTA/Air system could be used effectively in the acidic and neutral pH range. The cited authors suggested that the role of ultrasound in the Fenton process was both physical and chemical, based on the phenomenon of acoustic cavitation [99].

Cavitation could generate strong convection in aqueous media, because of ultrasonic vibration, micro-turbulence, and shockwaves. Ultrasounds can in fact produce large numbers of micro-bubbles in water, which grow and then collapse. Temperature and pressure inside adiabatically collapsing micro-bubbles can rise within a microsecond to levels of 4000–15,000 K and 100–5000 bar, respectively. As a result, hydroxyl radicals are generated from water vapor decomposition [34,99]. Man et al. proposed that in the ultrasound/ZVI/EDTA/Air system, O_2_ activation could occur at the ZVI surface or near it, with significant generation of H_2_O_2_ and free radicals that would then oxidize the contaminants. At the same time, a fraction of the contaminants could be removed from the solution upon adsorption onto the ZVI surface [99]. Because the PAH molecules are hydrophobic, they have high tendency to adsorb onto or around the ZVI surface. Therefore, PAHs might be removed more quickly by ultrasound/ZVI/EDTA/Air than other less hydrophobic organic compounds under the same conditions [99].

Fu et al. have studied the effect of pH (3–9) on selenite removal using ultrasound/ZVI. They found a definite pH dependence [34], which could be attributed to the pH trend of selenite speciation coupled with ZVI surface charge and Fenton reactivity. At pH ≥ 3 the dominant species of selenite is HSeO_3_^−^, whereas SeO_3_^2^^−^ ions are formed at pH ≥ 8. Selenite removal decreased at pH ≥ 8, due to the decline in both Fenton and adsorption processes. In fact, one can expect limited generation of ^●^OH radicals and a lower ^●^OH oxidation potential under alkaline conditions. Moreover, the negative surface charge of ZVI at pH ≥ 8.2 inhibits adsorption of negative HSeO_3_^−^ and SeO_3_^2^^−^ [34,100].

**Figure 3:**
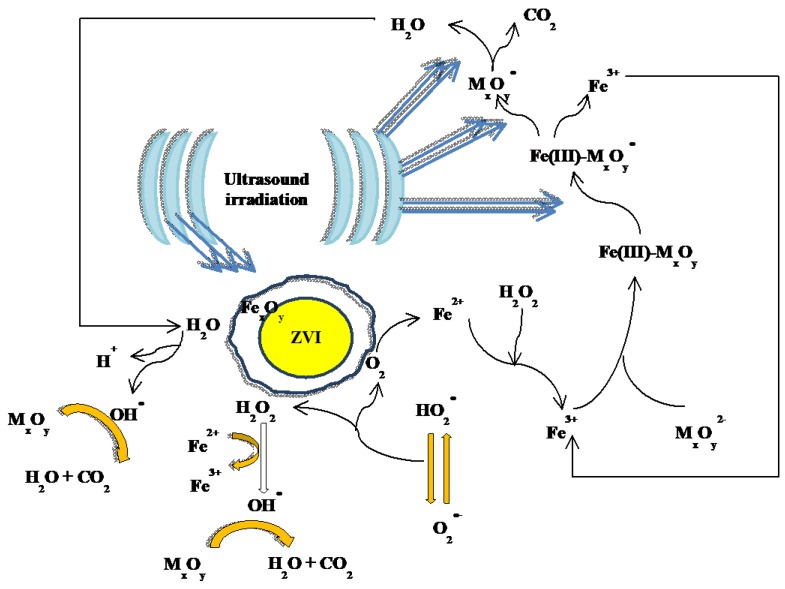
Schematic of a typical ultrasound-ZVI-Fenton mechanism (note that M_x_O_y_ represents a generic organic pollutant).

Taha et al. [101] and Weng et al. [5] have suggested that ultrasound radiation could not affect the production of Fe^2+^ by ZVI at pH above 4, due to the protection of the ZVI surface by a layer of iron oxides. The different findings of the two latter studies [5,101] compared to the other ones [34,100] could be attributed to the fact that Taha et al. [101] and Weng et al. [5] have used lower ultrasound intensity (0.12 vs. 0.3–1.08 W/cm^3^), lower degradation time (10 vs. 90 min) and lower amount of ZVI catalyst (0.5 vs. 5–30 g/L). It can be concluded that ultrasound radiation might extend the applicable pH range of Fenton-like processes to near-alkaline conditions, provided that sufficiently high ultrasound intensity and ZVI loading is used. A schematic of a typical ultrasound-ZVI-Fenton system is illustrated in Figure 3.

#### 3.2.4. Photo-Fenton Process

The Fenton reaction is often more efficient under irradiation conditions (photo-Fenton) than in the dark, because the photolysis of Fe(III) compounds yields Fe(II) and enhances Fe(III) recycling. Therefore, more Fe(II) can react with H_2_O_2_ and yield reactive transient species such as ^●^OH.

Santos-Juanes et al. have used a ZVI/H_2_O_2_/UV system and have reported a decrease with pH of the degradation percentage of p-nitrobenzoic acid, from 70% at pH 3.5 to <10% at pH ≥ 5 [1]. They have proposed that acidic pH and the presence of H_2_O_2_ could favor the oxidative photo-Fenton process, whereas at mild pH (especially if H_2_O_2_ was not added) the Fenton reaction was inhibited and the prevailing pathway was substrate reduction by ZVI [1]. Minella and coworkers have reported that ZVI was inactive in a UV-A/H_2_O_2_ system for the degradation of phenol at pH > 5, but the same system in the dark was already inactive at pH > 4 [101]. Rahim Pouran et al. have found that the ZVI efficiency in the photo-Fenton process decreased at pH > 4, due to precipitation of ferric hydroxide that reduced radiation transmission [102]. Likewise, Grcic et al. have shown that the specific (surface-area normalized) reaction rate constant for dye oxidation in wastewater using a UV-C/ZVI-Fenton system was 0.020, 0.019 and 0.012 L min^−1^ m^−2^ at pH 3.5, 4 and 5, respectively [7]. They suggested that at pH 2.5–5 the generation of ^●^OH radicals could take place through photolysis of the [Fe(OH)]^2+^ complexes (Equation (10)), in addition to the typical Fenton reaction (Equation (11)) [103].

[Fe(OH)]^2+^ + *hν* → Fe^2+^ + ^●^OH (10)

Fe^2+^ + H_2_O_2_ → [Fe(OH)]^2+^ + ^●^OH (11)

**Figure 4:**
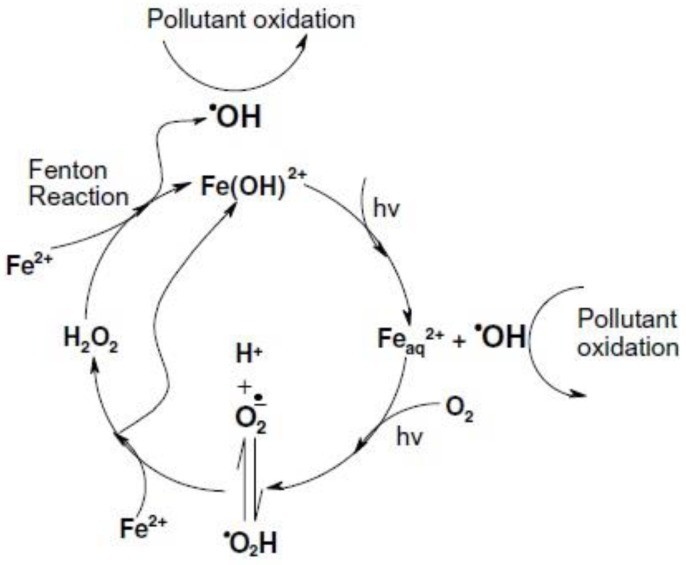
Model of photo-Fenton mechanism using iron-based catalysts. Reproduced with permission from Reference [104].

Because Fe(II) is oxidized to Fe(III) in the reaction with H_2_O_2_ (Equation (11)), the cycle can continue [103]. This cycle is anyway restricted in neutral and alkaline pH conditions due to two reasons: (1) generation of less reactive species like ferryl as the pH increases, instead of highly active ^●^OH radicals; (2) limited leaching of Fe species from oxide coating surfaces at pH > 5, which prevents the occurrence of photoactive [Fe(OH)]^2+^ [101]. It can thus be concluded that the ZVI/H_2_O_2_/UV system may be efficient up to circumneutral conditions, but not at alkaline pH. However, some studies have shown that a process set-up with physically separated compartments of ZVI corrosion and photo-Fenton reaction could enhance the degradation of hardly oxidized contaminants, even at neutral pH values [1]. In the first stage of such systems, contaminants are removed via adsorption onto the ZVI surface or are modified to produce more labile compounds, which are then oxidized in the subsequent photo-Fenton stage. In addition, iron cations leached to the solution in the first stage could further participate in the photo-Fenton process. Moreover, in a combined ZVI/H_2_O_2_/UV system, light scattering could occur due to the presence of the solid catalyst and it would decrease the amount of available photons for photochemical reactions [1]. An illustration of the photo-Fenton mechanism using iron-based materials is shown in Figure 4.

#### 3.2.5. Microwave-Assisted Fenton-Like Process

**Figure 5:**
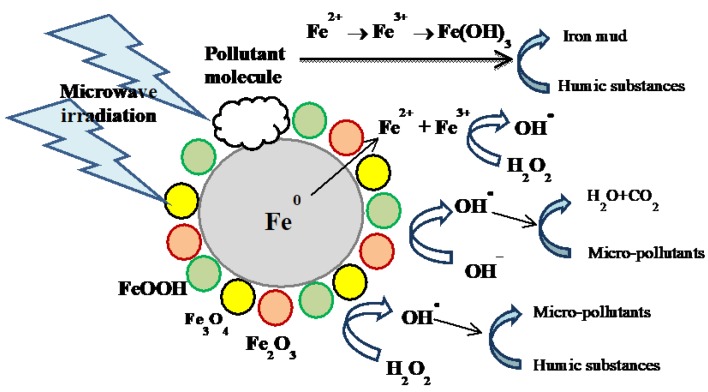
Typical microwave (MW)-ZVI-Fenton system. Reproduced with permission from Reference [17].

There are few studies in the area of microwave-assisted Fenton reaction using ZVI as a catalyst. Chen et al. have implemented microwave irradiation (MW) coupled with ZVI/H_2_O_2_ to treat concentrated landfill leachate [17]. According to BET analysis, the used ZVI had average pore size of 2–30 nm and specific surface area of 0.844 m^2^/g before the reaction. The average pore size changed to 2–50 nm after the reaction, and the authors proposed that microwave irradiation could modify the ZVI surface to produce larger pores, which are more efficient for degradation processes [17]. Zhang et al. have observed that in a ZVI/H_2_O_2_/MW system, the reaction rate constant decreased from 0.05 to 0.01 min^−1^ when the initial pH increased from 2 to 8 [105]. They related this result to the formation of iron-based colloids in alkaline conditions, which undergo coagulation phenomena and slow down pollutant removal [105]. Lee et al. have proposed that MW radiation may be absorbed unequally by ZVI particles, with formation of local hot spots having much higher surface temperature (435 °C) than the surrounding zones [106]. The temperature increase could induce higher ZVI reactivity and enhance the release of Fe^2+^ ions into the solution. In addition, under MW irradiation the mass transport accelerates at all pH values, because of an increase in the average kinetic energy and thermal motion of the molecules [107]. Hong et al. have suggested that not only strongly acidic conditions, but also strongly alkaline conditions (pH ≈ 12) could sharply enhance Rhodamine B decomposition in a H_2_O_2_/MW system. They found that H_2_O_2_ was decomposed into O_2_ in the presence of MW irradiation at pH 12, and O_2_ was then involved in substrate oxidation [108]. Moreover, some organic compounds such as phenols are in the anionic state at alkaline pH, where they absorb microwave radiation to a larger extent than at other pH values. Interestingly, pollutant degradation with H_2_O_2_/MW behaved differently from ^●^OH oxidation [108]. Remya and Lin have reported that carbofuran degradation by ZVI/H_2_O_2_/MW at 80 °C increased by increasing pH from 2 to 10 [109]. From the results of their study, 99–100% degradation of carbofuran was achieved after 60 min at pH 6. By increasing the pH to 8, the time needed for complete carbofuran decomposition decreased to 10 min. The cited authors suggested that the higher efficiency of ZVI at alkaline pH was probably due to radical oxidation processes, i.e., the transformation of OH^−^ anions into hydroxyl radicals due to the loss of electrons at higher temperature in the presence of MW [109]. Liu and coworkers have reported that the removal rates of 4-nitrophenol using a ZVI/MW system at ambient temperature were almost equal at pH 3 and 7 [110]. The same result has been observed by Jou for the removal of pentachlorophenol by ZVI/MW in water [111]. In the presence of MW, Fe^0^ and pentachlorophenol (C_6_Cl_5_OH) formed Cl^−^ and Cl_2_, which reacted with water vapor to produce HCl. The acid dropped the solution pH and intensified ZVI corrosion [111,112,113,114]. Although further studies are required to better understand the behavior of ZVI/H_2_O_2_/MW systems, generally it can be concluded that the addition of MW to ZVI/H_2_O_2_ enhances the degradation rate of contaminants at pH ranges from acidic to alkaline. However, the reaction pathway could substantially depart from a typical Fenton process. Moreover, the power consumption of microwave is always a concern in practical applications [93]. A typical microwave-ZVI Fenton system is shown in Figure 5.

## 4. Conclusions

In summary, pH significantly affects the ZVI performance for contaminants removal in ZVI-based heterogeneous Fenton processes. High degradation efficiencies are usually obtained in the pH range 3–4, which is mainly attributed to acceleration of iron corrosion, dissolution of the passive oxide layers on the ZVI surface, and efficient generation of hydroxyl radicals. In contrast, neutral and alkaline pH values decrease the ZVI-Fenton efficiency due to Fe(III) precipitation (or lack of solubility) and less effective ^●^OH production. A decrease of the solution pH below 3 could be detrimental to degradation, because it causes fast dissolution of ZVI particles, leads to an excessive accumulation of hydrogen bubbles at the ZVI interface, or both. These phenomena may decrease the available reactive surface area for contaminants removal. In addition, the solution pH alters the surface charge distribution of iron hydroxides/oxides, causes ionization of weak acid or bases (depending on their pK_a_ values), and affects solubility, speciation, as well as complex formation tendency of metal or metalloid contaminants. For example, increasing the solution pH to moderately basic conditions can favor the removal of some metal cations (e.g., Cu^2+^, Cd^2+^, Zn^2+^, Co^2+^) by enhancing their adsorption to iron oxides that coat the ZVI surface, or their precipitation as metal hydroxides. At pH values below the point of zero charge (pH_PZC_), the surface of ZVI is positive and it may attract negatively charged pollutants (e.g., Cr(VI) and As(V)).

Fenton-like processes, by addition of external energy to the heterogeneous Fenton reaction, can increase the ZVI performance to remove contaminants in a wide range of pH values. Moreover, the regeneration of ferrous ions can be accelerated. The combination of heterogeneous Fenton with a weak magnetic field, UV irradiation, ultrasound or microwave irradiation looks promising, although such techniques might be more difficult to apply in large-scale operations than in the laboratory. The generation of oxidizing radicals in Fenton-like processes may be almost independent of the solution pH in some cases, but ZVI corrosion is still influenced by pH. Many studies have shown the high performance of ZVI at acidic and neutral pH values in the framework of Fenton-like processes, but additional work is required to investigate the effect of strong alkaline conditions (e.g., pH values of 11–12) on the efficiency of ZVI-Fenton-like systems. Interestingly, in the context of industrial applications, other wastewater treatment methods such as filtration and biological treatment can also be coupled with Fenton-like techniques to achieve complete removal of organic pollutants. 

## Figures and Tables

**Table 1:** Overview of works done in the area of heterogeneous Fenton using ZVI as catalyst.

#	Target Compound	Experimental Conditions	Remarks	Ref.
1	Methylene Blue (MLB) Methyl Orange (MO)	ZVI: 0.05g; dye volume: 50 mL; initial contaminant concentration: 50 mg/L; H_2_O_2_ (10%): 5 mL; pH = 3	Degradation percentages: MLB: >80% in 5 min; MO: 80% in 1 h.	[84]
2	Orange II	ZVI: 0.02 g/L; initial contaminant concentration: 58 mg/L; H_2_O_2_ (28 *w*/*w*%); pH = 2–9	39, 52, 38, 22, 16, 16, 11, and 10% removal in 10 min at initial pH value 2,3,4,5,6,7,8,9, respectively.	[85]
3	Amoxicillin (AMX) COD	ZVI: 500 mg/L; AMX 50 mg/L; H_2_O_2_ 6.6 mM; pH = 3; temperature 30 °C	86.5% removal of AMX; 71.2% of COD removal in 25 min.	[86]
4	Norfloxacin (NOR)	ZVI: 100 mg/L; H_2_O_2_ concentration: 20 mmol/L; pH = 4; temperature 35 °C	90% NOR removal in 40 min.	[77]
5	4-chloro-3-methyl phenol (CMP)	ZVI: 0.5 g/L; CMP concentration: 0.7 mM; H_2_O_2_: 3 mM; pH = 3–6	99% CMP removal at pH of 3 and 6 in 1 and 15 min, respectively.	[87]
6	Pentachlorophenol (PCP)	ZVI: 15 mg; PCP concentration: 50 mg/L; H_2_O_2_: 0.5%; pH = 3; temperature 30 °C	90% PCP removal in 30 min.	[80]
7	4-chlorophenol (4CP)	ZVI: 1 g/L; 4CP concentration: 100 mg/L; H_2_O_2_: 0.5%; temperature 20 °C	100% 4CP degradation at pH of 3 and 4 in 8 and 30 min, respectively. 12.5% H_2_O_2_ decomposition at pH 6.5 and 70% at pH 5 in the same conditions.	[88]
8	Cr (VI), TOC, COD, Phenol	ZVI/H_2_O_2_ (*w*/*w*):0.5, 0.75, 1, 1.25, 1.5; H_2_O_2_/COD (*w*/*w*):0.5, 0.7, 1; pH = 2, 2.5, 3, 3.7, 5; temperature: 15, 20, 30, 35°C.	At H_2_O_2_/COD (*w*/*w*):0.5, ZVI/H_2_O_2_ (*w*/*w*):0.75 and pH = 3, there was a total removal of Cr(VI) as well as TOC, COD and phenols degradation efficiency up to 70, 73 and 88%, respectively.	[89]
9	Methyl tert-butyl ether (MTBE)	ZVI: 250 mg/L; MTBE concentration: 1000 µg/L; H_2_O_2_: MTBE (molar ratio) = 220:1; pH = 3, 4, 7.	MTBE removal of 99, 96, 72% at pH values of 4, 7, 3 respectively in 24 h.	[90]
10	Phenol	ZVI: 1 g/L; phenol concentration: 100 mg/L; H_2_O_2_ concentration: 1, 5, 10, 25, 50, 100 mM; pH = 2.5.	80% and 100% phenol degradation by 5 and 10 mM H_2_O_2_, respectively.	[91]
